# Investigation of metal ion binding biomolecules one molecule at a time

**DOI:** 10.3389/fchem.2024.1378447

**Published:** 2024-04-12

**Authors:** Micaela de la Torre, Adam Pomorski

**Affiliations:** Department of Chemical Biology, Faculty of Biotechnology, University of Wrocław, Wrocław, Poland

**Keywords:** metal ions, single molecule methods, biomacromolecules, biophysics, structural biology

## Abstract

Metal ions can perform multiple roles ranging from regulatory to structural and are crucial for cell function. While some metal ions like Na^+^ are ubiquitously present at high concentrations, other ions, especially Ca^2+^ and transition metals, such as Zn^2+^ or Cu^+/2+^ are regulated. The concentrations above or below the physiological range cause severe changes in the behavior of biomolecules that bind them and subsequently affect the cell wellbeing. This has led to the development of specialized protocols to study metal ion binding biomolecules in bulk conditions that mimic the cell environment. Recently, there is growing evidence of influence of post-transcriptional and post-translational modifications on the affinity of the metal ion binding sites. However, such targets are difficult to obtain in amounts required for classical biophysical experiments. Single molecule techniques have revolutionized the field of biophysics, molecular and structural biology. Their biggest advantage is the ability to observe each molecule’s interaction independently, without the need for synchronization. An additional benefit is its extremely low sample consumption. This feature allows characterization of designer biomolecules or targets obtained coming from natural sources. All types of biomolecules, including proteins, DNA and RNA were characterized using single molecule methods. However, one group is underrepresented in those studies. These are the metal ion binding biomolecules. Single molecule experiments often require separate optimization, due to extremely different concentrations used during the experiments. In this review we focus on single molecule methods, such as single molecule FRET, nanopores and optical tweezers that are used to study metal ion binding biomolecules. We summarize various examples of recently characterized targets and reported experimental conditions. Finally, we discuss the potential promises and pitfalls of single molecule characterization on metal ion binding biomolecules.

## 1 Introduction

**FIGURE 1:**
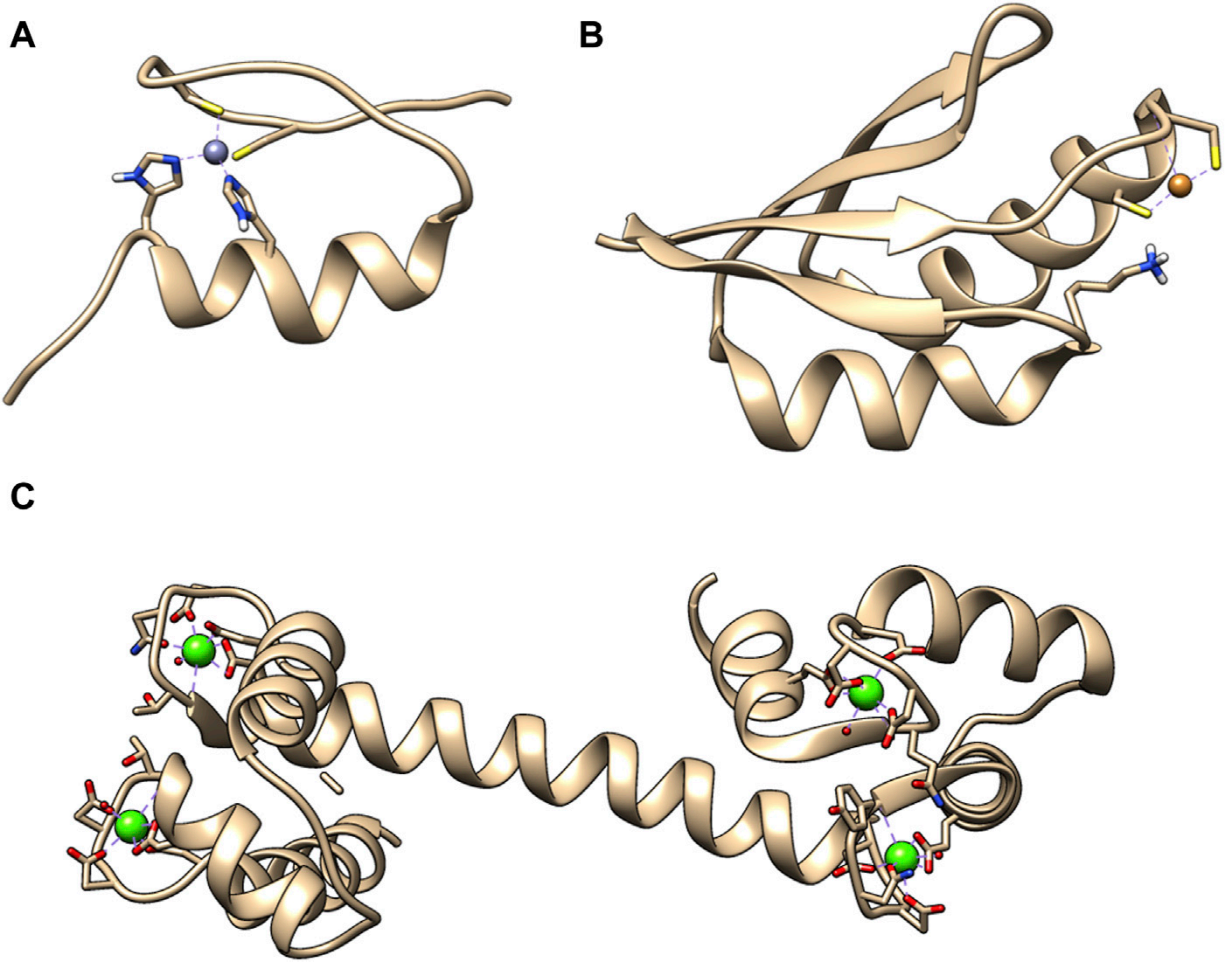
Exemplary 3D structures of proteins binding metal ions. **(A)** Zinc finger domain from transcription factor SP1F2 with bound Zn (II) (PDB: 1SP2). Zinc finger domains in most cases are responsible for transcription factor binding to the DNA or RNA. **(B)** Human metallochaperone ATOX1 (HAH1) loaded with Cu (I) (PDB: 1TL4). It is involved in the copper homeostasis. **(C)** Human calmodulin that binds 4 Ca (II). It is a protein that relays calcium signals to enzymes or channels that are sensitive to it (PDB: 1CLL).

Metal ions are crucial for the cell function and they perform multiple roles, from structural elements to signaling molecules. Only in humans, approximately one-third of the whole proteome binds metal cations (McRae et al., 2009). Furthermore, the number of diseases that have been characterized by metal imbalance in cells has steadily increased year by year, like in Alzheimer’s and Parkinson’s disease, which involve accumulation of metal ions in brain tissue (McRae et al., 2009). A general division can be made for metal ions that are abundant and in large concertation, e.g., Na^+^ and those, which cellular concentration is tightly controlled such as Ca^2+^, Zn^2+^, and Cu^+/2+^. For them usually the total concentration in cell is higher, then concentration available for metal ion binding proteins. This also led to the evolution of specialized binding site for those metal ions. Zn^2+^ ions are mostly known in connection with zinc finger DNA binding domains. This is not surprising as more than half of all predicted transcription factors utilize ZF to interact with genomic targets (Lambert et al., 2018). Ca^2+^ is commonly mentioned as a building material for bones and teeth. However, equally important is the calcium dependent regulation of cardiac myocytes function (Smith and Eisner, 2019). Cu^+/2+^ is commonly associated with superoxide dismutase, a key enzyme in protection against oxidative damage (Lewandowski et al., 2019). There are numerous research papers, reviews and book chapters describing the importance of Ca^2+^, Zn^2+^, and Cu^+/2+^ for protein activity, as well as cell function in physiological and pathological states, such as cancer (Choudhary et al., 2018; Kim and Lee, 2021; Ge E. J. et al., 2022). Figure 1 presents structures of example metal ion binding proteins.

Each year there are new discoveries related to those metal ions and their binding proteins. Among the most recent discoveries we learned that interaction between S100B protein, a potential marker of neurodegenerative disease with the neuronal calcium sensor protein, neurocalcin delta, is regulated by Ca^2+^ both *in vitro* and in living cells (Zhang et al., 2021). Zn^2+^ was found to be a potential protective factor during vascular calcification, which is related to increased cardiovascular mortality in diabetic patients (Henze et al., 2021). In 2022 it was discovered that cell death induced by excess copper proceeds through previously unknown mechanisms related to mitochondrial respiration, providing further evidence for evolutionarily conserved homeostatic mechanisms (Tsvetkov et al., 2022). Those discoveries highlight that there are still elements of the metalloproteome that has not been discovered.

To properly characterize those metal ion binding proteins several special considerations must be made. Protein can have a distinct function in the metal free (apo) or metal bound (holo) form. Moreover, Irving–Williams’s series, which describes relative stabilities of complexes formed by transition metals, shows the potential of some metal ions, like copper to occupy zinc sites, which can affect the biomolecule behavior. In such cases presence of excess of metal ions can lead to artifacts and improper conclusions. Therefore, numerous specialized approaches were developed, including competition-based experiments that utilize various spectroscopic methods to report the binding (Pomorski et al., 2013; Miłoch and Kręzel, 2014). However, even considering the low detection range for fluorescence the sample consumption is relatively high. Also, it is possible to observe the system only when it is equilibrated, which can take hours or days. There is only one review from 2010 that summarized the single molecule experiments where the target was a metal ion binding protein (Chen et al., 2010). It mostly focuses on the metal ion dependence of binding of MerR transcription factors metaloregulators from bacteria to the DNA. There are also a few reviews that discuss specific achievement of single molecule methods to characterize a metal ion binding protein. For example, single molecule studies on Neuronal Calcium Sensor-1 (NCS-1) provided crucial information on apo Ca^2+^ and Mg^2+^ bound states, revealing a complex folding mechanism with rugged and multidimensional energy landscape (Choudhary et al., 2018). Here we make a broad summary of studies on metal ion binding biomolecules utilizing various single molecule methods.

## 2 The promises and pitfalls of the single molecule methods analysis of metal ion binding biomolecules

**FIGURE 2:**
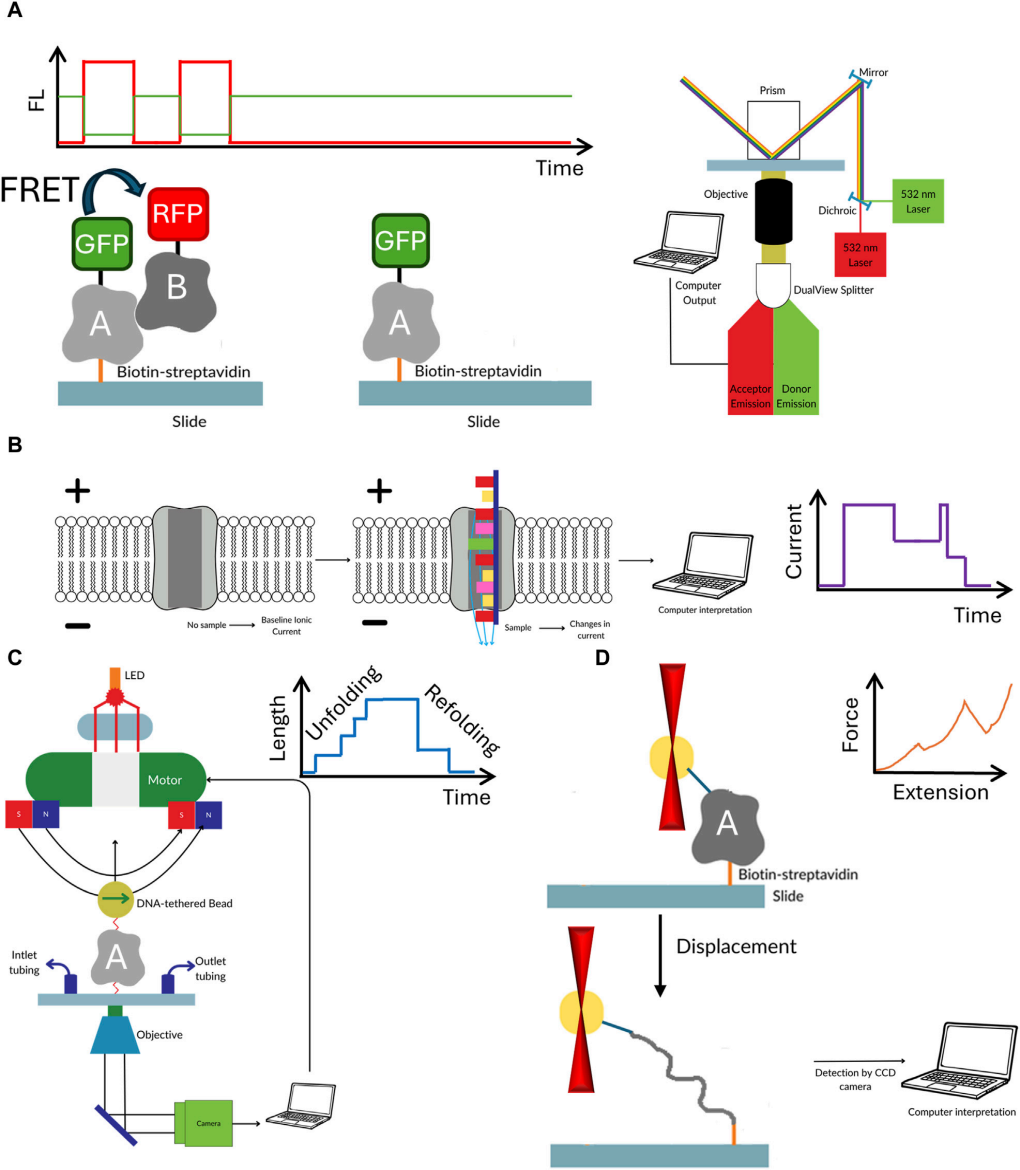
The workflow of the discussed single molecule methods. Single molecule FRET **(A)** requires the biomolecules to not only be fluorescently labelled with FRET pair, but also have biotin for easy immobilization of to the surface of microscope slide. In nanopores **(B)** the electric current passing through the pore is temporarily blocked by the passing biomolecule. Though labelling is not required in this method often different tags and moieties are added to both the pore or target molecule to enhance the sensitivity. In optical **(C)** or magnetic tweezers **(D)** the target biomolecule is suspended between beads which are manipulated either by laser beam or magnetic field. The target must be extensively modified by to attach spacer molecules at both ends of the studied biomolecule. While in optical tweezers usually one molecule is studied, the magnetic tweezers offer the ability to observe several molecule at the same time.

The key advantage of single molecule methods, such as single molecule FRET, nanopores or optical/magnetic tweezers is that they remove the need for synchronization (Roy et al., 2008; Koch and Shaevitz, 2017; Schmid and Dekker, 2021). Each molecule is analyzed individually, therefore no ensemble averaging, that often accompanies the bulk measurements, is observed. This feature masks the underlying molecular dynamics because the measured signals are the unsynchronized average of the contributions of every molecule in the sample (Tinoco and Gonzalez, 2011). For example, in case of protein—DNA interaction the bulk FRET signal provides an information that there was a binding, whereas single molecule experiments can determine how exactly the protein was able to find the correct interaction site. Finally, the sample consumption is extremely low, and one could argue that the detection limit is just a single molecule of the target of interest. As with all methods, apart from above mentioned advantages single molecule methods have some disadvantages (see Table 1; Figure 2). Some of them require multiple site-specific reactions to tether them to surface or fluorescently labeled. The nanopore size must be adjusted to target size. Additionally, methods based on fluorescence suffer from photobleaching, though this can be partially mitigated by application of photostable dyes and oxygen scavengers (Aitken et al., 2008; Zheng et al., 2014). Observation of protein-protein interaction also requires rigorous surface passivation to limit nonspecific binding to the surface to prevent analysis of nonspecifically bound molecules (Chandradoss et al., 2014).

**TABLE 1:** Advantages and disadvantages of the different single molecule methods.

	Advantages^a^	Disadvantages
Single molecule FRET	Signal from up to 4,500 molecules can be collected at once	Need to utilize several site specific modifications for labelling
		The distance of FRET donor and acceptor has to be optimal for the effect to occur
Nanopores	No labeling required^b^	The pore size has to be perfectly matched to the molecule size
	Low cost	
Optical tweezers	Provides a constant force	Photodamage and thermal denaturation caused by the laser
	The force can be applied to moving objects	Only a single molecule can be measured at a time^c^
Magnetic tweezers	Provides non-contact force	Low temporal and spatial resolution
	Low cost	
	Multiple molecule can be measured at once	

^a^ The common advantages of all the methods are single molecule sensitivity and no need for synchronization prior to the experiment.

^b^ Various tags or designer molecules are sometimes conjugated to the target to enhance the detection.

^c^ Optical trapping of multiple objects is possible.

The above mentioned advantages and disadvantages of single molecule methods can be universally attributed to any type of studied biological molecules. What about metal ion binding ones? Certainly a promise of single molecule measurements is the ability to characterize targets that are very difficult to produce. One such example would be membrane bound proteins, including receptors and transporters. Proteins anchored in liposomes are routinely analyzed using various single molecule fluorescence methods e.g. (Tutkus et al., 2018). An interesting target could be also molecules derived directly from studied organism. We often study human metalo proteins produced in *Escherichia coli*. Though this method usually provide us with medium/large amounts of target protein, it does not have the post-translational modifications. There are multiple evidences of post-translational modifications (PTMs) influence on binding of the metal ion by proteins (Mathis and Barrios, 2021; Nolan and Peet, 2023).

There are also several pitfalls of the single molecule methods that we should be aware of when analyzing the molecules. The first concern should be the presence of the metal ion in the biomolecule during the experiment. This is directly correlated to dissociation constant of the complex. Though Cu^+/2+^ tends to form high affinity complexes, some of the Zn^2+^ regulatory sites or Ca^2+^ binding sites are in the range of *K*_d_ between 10^–7^ and 10^–4^ M. Single molecules might not be a method of choice for the latter. In bulk methods the value of *K*_d_ is obtained from concentration based calculation obtained by measurement of the complex at equilibrium. In case of single molecule methods values of *k*_on_ and *k*_off_ are obtained. By dividing *k*_off_ by *k*_on_ the value of *K*_d_ can be calculated. However, due to popularity of the bulk measurements the equilibrium calculated *K*_d_ values are more easy to obtain from publications and databases then the kinetic constants. However, they are crucial in single molecule experiments. In case of single molecule fluorescence acquisition by camera, the complex must be formed for at least several hundred milliseconds, so considering the standard data acquisition frequency of 10 frames per second enough data points can be acquired. Complex *k*_off_ value will determine the number or recorded events, however *k*_on_ is also important as the binding must occur before photobleaching occurs. Therefore, in practice in order to be able to record enough data points the complex *K*_d_ value should be at least submicromolar. Single molecule methods consume minimal amount of sample, typically sub µM or pM. This together with complex *K*_d_ should also be considered. For example, we have a nanopore based experiment, where there is a distinction of current blocking between free biomolecule and metal ion bound one. At 50 nM biomolecule concentration and 100 nM metal ion at equilibrium only 1% of total events is related to complex passing through the nanopore. A quick calculation reveals that *K*_d_ of such complex is 9 μM. Therefore in order to acquire statistically relevant amount of datapoints long term measurement would have to be performed. This is why in some reported experiments the metal ion was added in excess to the solution measured using single molecule methods (see below).

An interesting approach to keep the bulk concentration has been described for nucleosomes. Single molecule FRET readout was possible by labelling only a small portion of the total protein added to measurement chamber (Gansen et al., 2013). On the other hand establishment of the high affinity complex dissociation constant, which is needed to judge the metal ion occupancy in the target biomolecule within its cellular environment, would also require competition based experiments to observe metal ion free species. This issue is similar as when classical methods, e.g., UV-VIS spectroscopy is applied. The thought experiment described above assumes measurement in the solution. Several single molecule methods require anchoring of target molecule on the surface of measurement chamber. After immobilization the excess target is removed, shifting the equilibrium. Whether this would affect the metal ion-biomolecule complex measurement strongly relies on the *k*_off_ value. With short experiment one can expect majority of high affinity complexes to remain intact. Though control experiment must accompany to confirm such statement. Finally, the buffers and additives, e.g., Gloxy system utilized to minimize photobleaching during any type of single molecule fluorescence observation should also be taken in the account. So far such a study was not reported. Even low affinity metal ion binding reagents can become competition agents, since they can be present in 10^6^ concentration excess over studied the studied metal ion-biomolecule complex. Though single molecule has been used to observe a number of metal ion binding biomolecules the concerns described above are not always addressed in the experiment and controls.

## 3 Exemplary experimental conditions used for characterization of metal ion binding molecules at a single molecule level

Different single molecule methods have certain prerequisites regarding their measurement, which are related to having specific sample requirements, and varying concentrations of metal ions and of the biomacromolecule sample. In some studies, the metal ion is added in excess compared to the protein concentration, to ensure its presence in the protein, as discussed above. Although it should be mentioned here that, as described above, addition of excess metal ion can lead to binding to unspecific sites that can be transient or prolonged (Foster et al., 2022). An example of single molecule experimental conditions are presented in Table 2. On the other hand, some studies present that addition of excess metal ion during single molecule measurement is not necessary. For example, structural protein zinc binding sites have so high affinity that the metal ion can be bound when expressed and carried throughout purification. However, such conclusions have to be always supported by protein: metal ion ratio study utilizing, for example, ICP-MS. This approach was utilized in the study on O^6^-alkylguanine DNA alkyltranferase (AGT). After purification the molecule still possessed a 0.72 ratio of Zn^2+^:protein and was directly utilized for measurement without addition of Zn^2+^ ions to measurement buffer (Kono et al., 2022). The results, further discussed in Section 5 showed the Zn^2+^ affects the distance translocated by AGT on DNA.

**TABLE 2:** An overview of experimental conditions used in single-molecule characterization of metal ion binding proteins.

Method	Sample preparation requirements	Example experimental conditions		
		Target concentration	Metal ion concentration	Reference
Optical tweezers (including fluorescence OT)	Site specific attachment of protein to beads through linkages	single molecule is trapped	0–1 mM Ca^2+^	Ge et al. (2022b)
smFRET	Immobilization of sample to the coverslip through linkage	25–100 nM	100 µM Zn^2+^	Luo et al. (2021)
Nanopores	Highly purified sample	5 µM	0.03 µM–1.0 µM Cu^2+^	Wei et al. (2018)

## 4 Single molecule characterization of Ca^2+^ binding biomolecules

As mentioned previously, Ca^2+^ is an essential metal ion in the human body. The effect of this metal ion in the context of the von Willebrand Factor (VWF) was tested. The VWF is a plasma glycoprotein that mediates platelet adhesion along the endothelium. In addition, it has been linked to inflammation, angiogenesis, and even metastasis (Dhami et al., 2022). VWF factor has been the target of several single molecule studies over the years. In one of the most recent Löf and his team presented the unfolding and refolding data for this multidomain dimeric protein (Löf et al., 2019). Single molecule methods allow for observation of the unfolding steps, which cannot be distinguished in bulk measurements. The authors utilized single-molecule magnetic tweezers force spectroscopy, as previous studies obtained with atomic force microscopy suffered from limited force resolution, preventing full description of the unfolding process (Möller et al., 2016). The obtained data allowed to establish that A2 domain is stabilized by Ca^2+^ by increase in refolding by 2- to 6-fold and reducing unfolding. In previous study on VWF A2 by Jakobi et al. (2011), where optical tweezers were applied, a long-lived intermediate in the unfolding pathway in the presence of Ca^2+^ has been observed, which was not found in Löf’s study (Löf et al., 2019). However, this difference may not stem from the difference in set force accuracy or sensitivity. Jakobi et al. (2011) proposed that unfolding of VWF A2 proceeds via mechanically stable intermediate with non-native Ca^2+^ coordination. The measurements were performed in the presence of 2.5 mM Ca^2+^, whereas Löf et al. (2019) utilized more “physiologically relevant” buffer, as described in the original article, containing both lower amount of Ca^2+^ (1 mM) and 1 mM Mg^2+^. Additional study would be required to establish if this is the only cause of the difference in obtained results, however as discussed in in chapter 2 the presence of an excess of the studied metal ion over target biomolecule, leads to binding to non-physiological sites. This can have severe consequences for the interpretation of the data, as well as its biological relevance. Though it should be noted that both studies agree that the presence of Ca^2+^ causes marked acceleration of VWF A2 folding.

**FIGURE 3:**
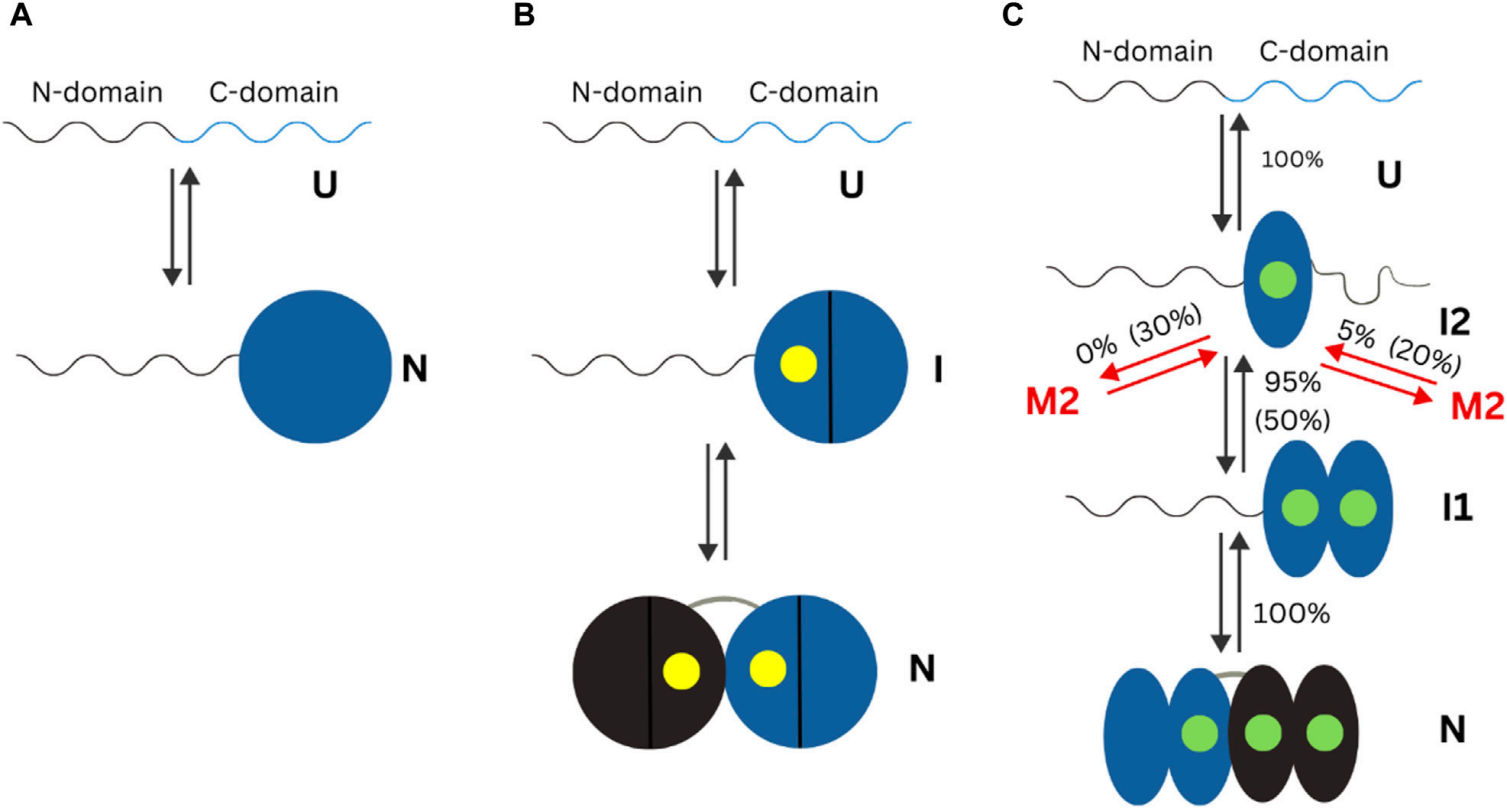
Schematic representation of the mechanisms of non-myristoylated NCS-1 in different ionic conditions measured using optical tweezers. In the present figure, “U” refers to the unfolded state of a protein. “N” refers to the folded (native) state of a protein. “I” indicates an intermediate state. **(A)** In the absence of divalent ions, the C-domain fluctuates between a folded and unfolded state, while the N-domain remains unstructured or loosely folded. **(B)** In presence of Mg^2+^ (presented as a yellow dot), the divalent ions bind first to the third EF domain, triggering the folding of the C-domain, and then to EF2, making the NCS-1 transit into its native state. **(C)** In the presence of Ca^2+^ concentrations, NCS-1 folds into a native state through a four-state process involving intermediates I1 and I2. Figure adapted from Choudhary et al. (2018).

Other protein domains have been studied under the context of their conformational or overall changes in the presence of different metal ions. The human neuronal calcium sensor-1 (NCS-1) is a two-domain EF-hand protein expressed mostly in neurons. NCS-1 transduces Ca^2+^ changes in neurons and is related to a broad number of neuronal functions, like voltage-gated Ca^2+^ channels and regulation of neurotransmitter release (Choudhary et al., 2018). Defective NCS-1 is damaging for cells, being linked to disorders like autism. The energetics of the transition from one conformation to another in NCS-1 is tightly regulated by binding divalent cations. Establishment of the conformational dynamics of all metal ion bound and free states is crucial to understand the molecular mechanisms underlying the biological functions of NCS-1. A 2018 mini review highlights, the state of knowledge on changes in the human neuronal calcium sensor-1 (NCS-1). Crucial data for elucidation of the metal ion importance came from studies where optical tweezers were used to study conformation of this protein at different concentrations of Ca^2+^ and Mg^2+^(Heidarsson et al., 2014; Naqvi et al., 2015). In the apo form of NCS-, the N-1 -domain is unstructured or loosely folded, while the C-domain is collapsed in a folded conformation. In presence of Mg^2+^, N- and C- domains acquire compact and mechanically resistant conformation that under tension loosens up and gain structure as separate units (Naqvi et al., 2015). At Ca^2+^ concentration of 0.5 µM, which is considered as physiological, only 5% of NCS-1 molecules misfold, but as the Ca^2+^ increases, so does the misfolded population and at [Ca^2+^] of 10 mM, one molecule out of 2 is lead to a non-native structure (Heidarsson et al., 2014). The differences in folding of apo or Ca^2+^ and Mg^2+^ bound NCS-1 found using single molecule methods are summarized in Figure 3.

Research on Ca^2+^ has also been focused on the extended synaptotagmins through the usage of optical tweezers. Extended synaptotagmins (E-Syts) are a group of proteins that mediate the exchange of lipids between endoplasmic reticulum and plasma membrane. Thanks to single molecule observation, it was possible to describe how Ca^2+^ is affecting E-Syt1 and E-Syt2 binding to the membrane (Ge et al., 2022). C2 domains are the most abundant membrane binding domains, and they are connected by disordered polypeptides which include synaptotagmins like E-Syts. It is also emphasized that the developed optical tweezer method can be applied to study interaction of proteins with multiple membrane binding domains, linked by disordered regions with membrane, which is incredibly difficult to ascertain using previously used methods. The effect of Ca^2+^ dependent membrane binding was characterized utilizing a flow control system that facilitated the change in metal ion concentration. In presence of 100 µM Ca^2+^, E-Syt1 showed three state transitions, same as in the presence of 10 µM. However, the former presented a reduced equilibrium force for C2CD transition. In the absence of Ca^2+^, the C2CD transition completely disappeared. The binding energy of both C2CD and C2E as a function of Ca^2+^ concentration was established. C2CD is undetectable in absence of Ca^2+^, increases in the range 0.1–10 µM Ca^2+^ and reaches saturation above 50 µM Ca^2+^. In the case of C2E, binding was Ca^2+^ independent as it does not contain a Ca^2+^ binding motif (Ge et al., 2022). Importance of Ca^2+^ has also been shown on a single molecule level for calerythrin. Calerythrin is a multi-domain, ribosome-bound protein, possessing four Ca^2+^ binding EF hands. Optical tweezers were used to study calerythrin folding on an actively translating ribosome and on the surface of stalled RNCs (Alexander et al., 2019). Previously, through nuclear magnetic resonance and circular dichroism studies it was suggested that the C domain folds more quickly than the N domain upon addition of Ca^2+^ (Aitio, 2001). However, as discussed above this data comes from an ensemble of molecules. The single molecule data revealed that the growing peptide is not equilibrated with its ensemble of accessible conformations (Alexander et al., 2019). Observation of Ca^2+^ binding is not limited to proteins. Heaton and Platt developed a sensor for this ion based on DNAzyme attached to a nanoparticle where the detection was achieved using nanopore based resistive pulse sensor (RPS) (Heaton and Platt, 2020). The reported limit of detection was 1 μM. In another approach it has been identified that Ca^2+^ flux around the nanopore caused retardation of translocation of both DNA and RNA (Wang et al., 2020). This phenomenon can be utilized not only as a new strategy of calcium sensing, but also a novel approach to detect the nucleic acids. Presence of Ca^2+^ made it possible to discriminate between DNA and RNA [poly (dA) vs. poly (rA)] as well as identify RNA homopolymers build by different bases (Wang et al., 2020).

## 5 Single molecule characterization of Zn^2+^ binding biomolecules

**FIGURE 4:**
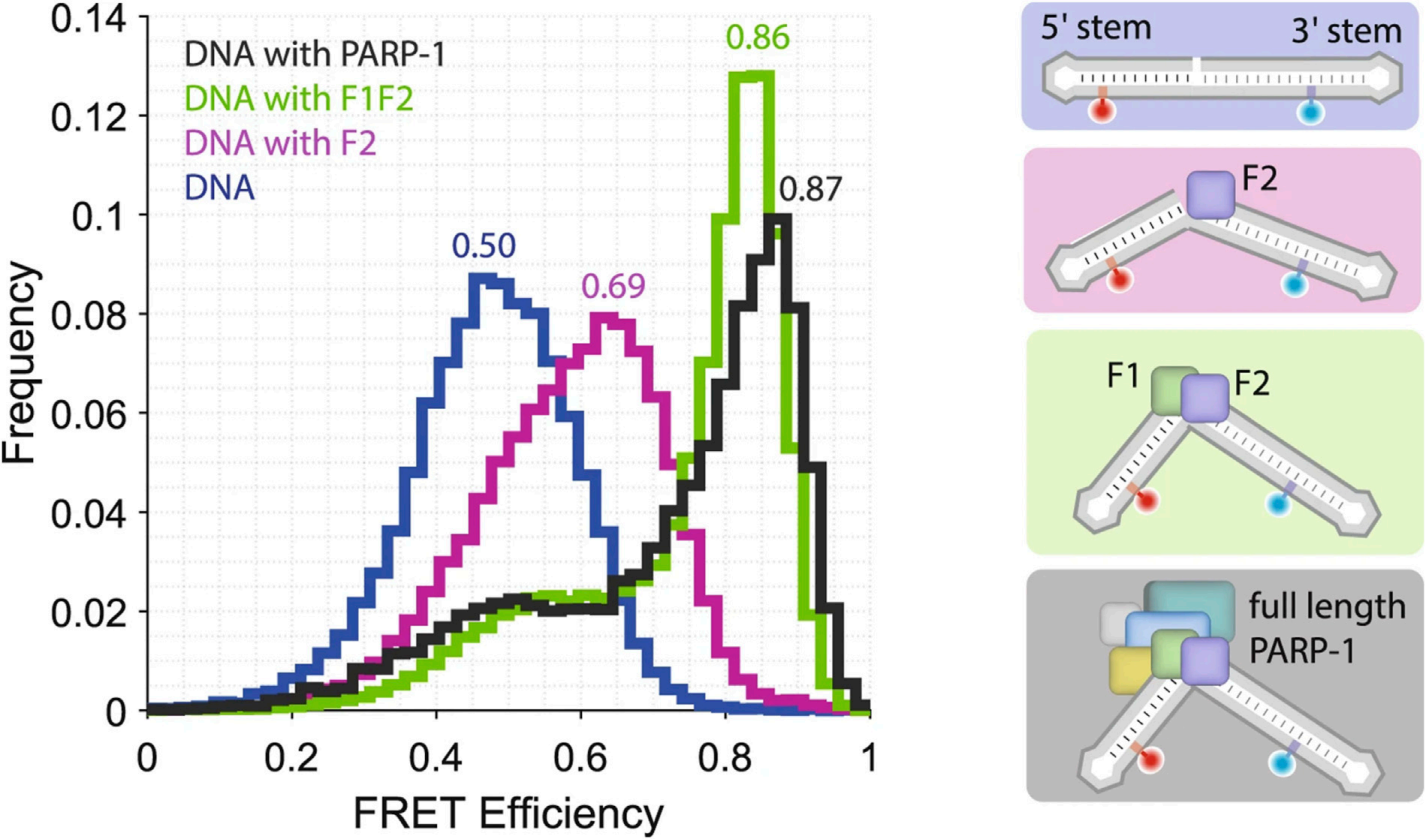
Single molecule FRET allowed to determine the role of the zinc fingers F1 and F2 from the PARP-1. smFRET efficiency histogram of nicked DNA (blue) and nicked DNA in the presence of either F2 (magenta), F1F2 (green) or full-length PARP-1 (grey here), together with cartoons illustrating the underlying conformations. Figure reproduced from (Sefer et al., 2022).

As described in the introduction, Zn^2+^ is most known as the metal ion in zinc finger domain (ZF). These structures have been used as a target of several single molecule studies. Fluorescence optical tweezers were used to understand the role of Zn^2+^ ion in the mechanism of the O^6^-alkylguanine DNA alkyltranferase (AGT) activity and mobility on DNA (Kono et al., 2022). Although it was shown that Zn^2+^ occupancy does not affect the AGT activity during lesion search, the single molecule analysis revealed for the first time that presence of Zn^2+^ affects the distance translocated by AGT on DNA and pointed to Zn^2+^ role in AGT cluster stabilization (Kono et al., 2022). Similarly, Rudnizky and co-workers investigated how Egr-1, a transcription factor possessing three zinc fingers, interacts with its target DNA sequence. Using optical tweezers, it was possible to determine that the complex is more easily dissociated if the interaction of third zinc finger (ZF3) is disrupted first (Rudnizky et al., 2018). Moreover, by combining optical tweezers with smFRET, folding of a small zinc finger domain known as ADR1a was studied. In contrast with the previous result, the study revealed that the domain may adopt alternative conformations when Zn^2+^ is missing, which were not found while performing the experiments in the presence of Zn^2+^ in concentration of 50 μM and 50 µM TPEN. These different alternative conformations can exhibit either lower or higher unfolding forces, as seen in the ribosomal tunnel (Wruck et al., 2021). Single molecule microscopy was used by Damon, and coworkers to establish the influence of Zn^2+^ on transcription factor dynamics (Damon et al., 2022). Their findings indicated that CTCF (CCCTC-binding factor), but not GR (glucocorticoid receptor), shows increase in the mean squared displacement and apparent diffusion coefficient when cellular Zn^2+^ coming from addition of ZnCl_2_ [30 µM] is chelated with Tris (2-pyridylmethyl) amine (TPA). Both of those proteins possess ZF domains. This suggests that CTCF is more dynamic when cellular Zn^2+^ is low. Both Zn^2+^ and TPA decreased the dwell times for CTCE. On the other hand, GR was largely unaffected by perturbation of Zn^2+^. These results show that some TFs are sensitive to change in the Zn^2+^ pool while others are not. Application of smFRET measurements allowed to determine the role of the zinc fingers F1 and F2 from the PARP-1 (Sefer et al., 2022). This protein is crucial in localization of single strand DNA breaks (SSB). It was found that the F2 alone can introduce a kink at the DNA nick, which is not present in isolated DNA when measured in the presence of 50 µM Zn^2+^ in buffer (Sefer et al., 2022). Further kinking at the nick occurs in the presence of F1F2. For details see Figure 4.

NMDAs (N-methyl-D-aspartate) are the main calcium permeable excitatory receptors in the mammalian central nervous system that play role in synaptic plasticity (Jewett and Thapa, 2022). Its receptor gating is complex, and has multiple open, closed, and desensitized states. The regulation of the NMDA receptor has been recently reviewed (Mony and Paoletti, 2023). However, there are unanswered questions about the conformations and energetics of the transmembrane domains as they relate to the gating states. By using smFRET, the energy landscape of the first transmembrane segment of the *Rattus norvegicus* NDMA receptor under resting and various ligand conditions was mapped (Dolino et al., 2017). The conformational states were examined when bonded to Zn^2+^, an allosteric inhibitor, to fully understand the gating motions of NMDA. Metal ion binds to the N-terminal domain of GluN2 subunits inducing cleft closure and reduction in channel activity. When compared to an open-channel blocker, Zn^2+^ bound state was more compact, which indicates differences for the two kinds of inhibitions. Importantly, Zn^2+^ bound form exhibits a similar number of transitions to other ligands conditions, despite being less active electrically and having an overall histogram reminiscent of the apo condition (Dolino et al., 2017). Similarly, single molecule FRET, among other methods, was used to show how Zn^2+^ affects different phenotypes of the NMDA receptors (Choi et al., 2013). It was found that 1 µM Zn^2+^ reduced current amplitudes in the receptor formed by wild type GluN1/GluN2B. However, when the receptor was formed by GluN1 and GluN2B mutant, where proline to alanine mutations were introduced in the region 1259–1356, and 1410–1482 the inhibitory effect of Zn^2+^ was significantly lessened. This phenotype, where the receptor is less sensitive to extracellular Zn^2+^, is highly reminiscent of that associated with Src kinase phosphorylation of GluN2B.

The Zn^2+^ binding domains are also important for the function of other proteins from various domains of life. For example, using smFRET, the conformational dynamics associated with DNA bending induced by HIV-1 nucleocapsid (NC) were studied. The experiments performed by Wang and coworkers utilized a series of DNA segments with different characteristics in presence of wide-type HIV-1 NC and NC mutants lacking N-terminal domain or zinc fingers (Wang et al., 2013). DNA bound with multiple copies of HIV-1 NC (nucleocapsid protein) shows multiple bent conformations. NC distorts the DNA local structures and bends it on a non-specific matter; the N-terminal domain and zinc fingers of NC are indispensable for this process to take place. Zn^2+^ is an essential element in all domains of life, nevertheless, the selective acquisition of it by *Streptococcus pneumoniae* from the infected eukaryotes is poorly understood. Zn^2+^ is known to induce minor changes in the global protein conformation of AdcA and stabilizes a highly mobile loop within its N domain. This loop region, in general, facilitates closure of the AdcAn binding site and is crucial for Zn^2+^ acquisition. A study by Luo et al. (2021) utilized smFRET to confirm findings from the crystal structure that Zn^2+^ (in concentration 100 µM Zn^2+^ + 1 mM EDTA, i. e., a metal ion buffer) does not induce conformational changes to AdcA. It has also been shown for the first time that Zn^2+^ bound with high affinity to AdcA N-terminal domain can be readily released. These results are concluded from seeing that the crystal structure of Zn^2+^ bound truncated AdcAn did not reveal any conformational differences by comparison with the N-terminal domain of the full-length, wild-type protein. Similarly, the Zn^2+^ bounded structure of AdAc did not reveal conformational differences. The lifetime of AdcAn-Zn^2+^ complex is short (Luo et al., 2021).

Single molecule characterization of the Zn^2+^ binding to biomolecules is not only limited to proteins. A combination of DNAzyme, DNA supersandwich structures and biofunctionalized nanopores allowed for development of a sensitive Zn^2+^ sensor (Liu et al., 2016). The device offers a detection limit of 1 nM, with great discrimination from Cu^2+^, Hg^2+^, and Pb^2+^.

## 6 Single molecule characterization of Cu^+^/Cu^2+^ binding biomolecules

Cu^+^/Cu^2+^ is an indispensable trace ion which plays an extremely important role in the biochemistry of every living organism. To understand how metalloregulators and DNA interact to maintain an appropriate metal ion level, Chen et al. (2013) developed engineered DNA Holliday junctions as reporters for metalloregulator-DNA interactions. Protein-induced DNA bending motion, which is essential for transcriptional activation, was detected by smFRET using DNA HJs. To avoid glass-protein interactions, a nanovesicle was used. The DNA HJs were used to study the DNA of two Mer-R family metalloregulators, PbrR691 and CueR, a Cu^+^ responsive regulator from *Escherichia coli*. Upon inducing a metalloregulator, the protein binding cause changes in the engineered HJ. This bending is essential for transcriptional activation. This was detected by comparing the FRET efficiency of each conformation. Similarly, azurin, an extensively studied protein carrying a copper ion was used as a model system. A reason why it was chosen is its relatively easy detectability through smFRET (Chen et al., 2010). When the Cu site contains an oxidised form of Cu (Cu^2+^), it displays a strong absorption in the 550–650 nm. This strong signal disappears when the Cu site is reduced. This change modulates the fluorescence properties of a FRET pair. These evaluations involved a protein amount in the range of a few nM. Through these experiments, it was determined that the fluorescence of a dye coupled to a protein can be strongly affected by a change in oxidation state of the protein (Chen et al., 2010). Single molecule observation of Cu^+^ dependent Hah1-MBD4 interaction dynamics provides further information on how the protein interactions are coupled to the metal ion transfer process. Even though MBD4 notoriously interacts with Hah1 directly for Cu^+^ transfer, it was shown that Hah1 forms complexes with MBD4 (WDP) despite the absence of Cu^+^ (Schmauder et al., 2005). Another important protein machinery in which copper is involved is the intracellular metal ion trafficking, which transports copper to its functional locations using copper chaperones. The copper chaperone Hah1 delivers Cu^+^ to MNK (Menkes disease protein), or the WDP (Wilson disease protein), which the purpose of incorporation into copper-requiring enzymes or for efflux. WDP and MNK are large, multidomain proteins, which on their N-termini have six metal ion binding domains. Hah1, on the other hand, is a single-domain cytoplasmic protein. When Hah1 and MBD interact, Cu^+^ can be transferred between them through a thiol ligand exchange mechanism. The quantification of these interactions, specifically on the dynamics of copper chaperones, has not been intensively researched. In 2010 Benítez et al. (2010) studied the interactions between MBD4 of WDP and Hah1. Through the usage of smFRET and considering that one Cu^+^ was added per Hah1-MBD4, it was determined that regardless of their metalation state, Hah1 and MBD4 can form two complexes that interconvert dynamically. This is an advantageous feature for their Cu^+^-transfer function. When only one of the proteins has metal ion bound, Cu^+^ is bridging both molecules at the protein interface via cysteines from both proteins, stabilizing the complex. Cu^+^ induced stabilization of interaction complexes was not observed when binding cysteines in Hah1 are mutated to serine residues to eliminate its Cu^+^ binding ability or when both proteins have Cu^+^ bound (Benítez et al., 2010).

**FIGURE 5:**
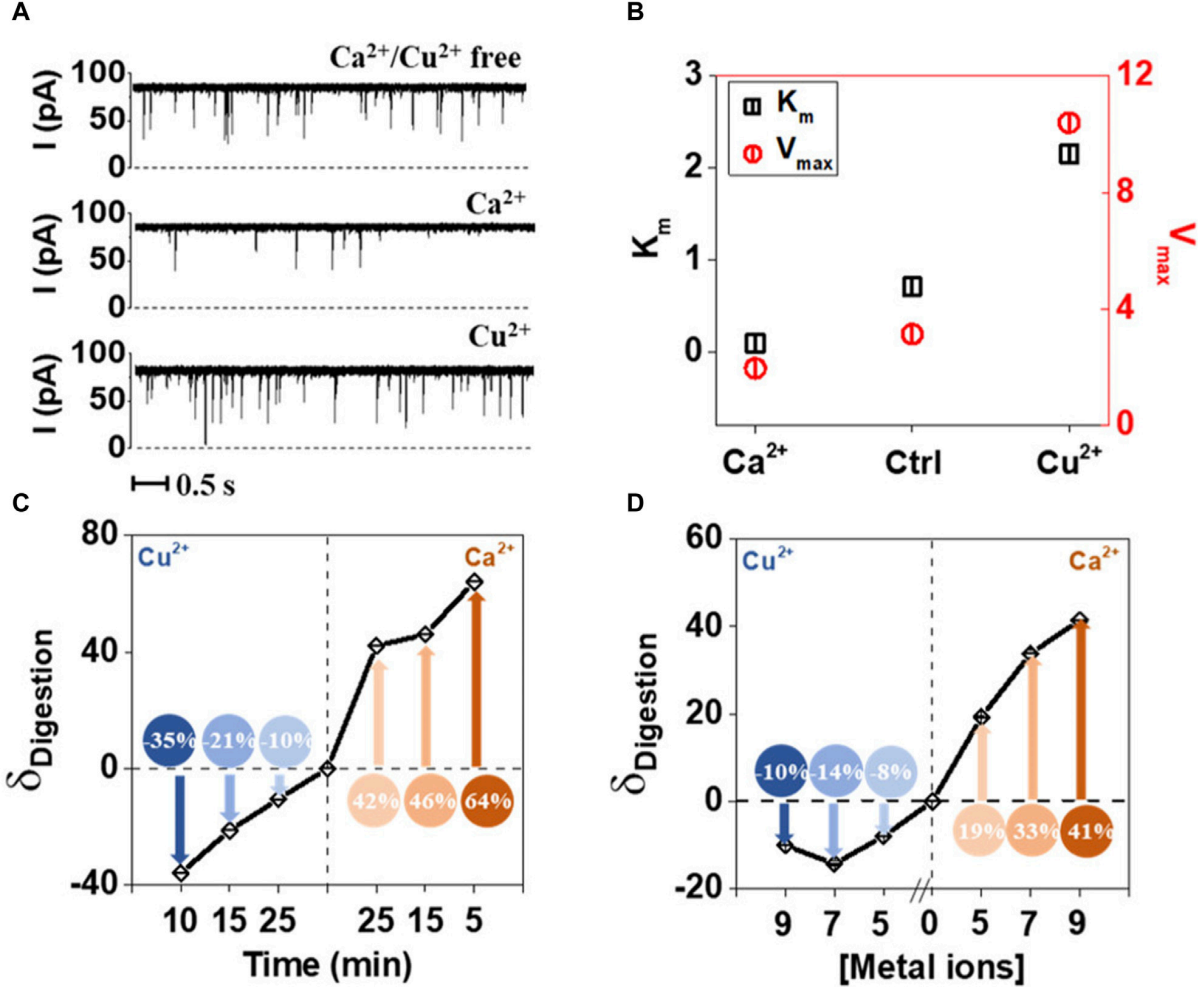
Effect of Ca^2+^ and Cu^2+^ on the tryptic proteolysis of Tat peptide measured using nanopores. **(A)** Electric current traces of digestion of Tat by trypsin in the absence and presence of Ca^2+^ or Cu^2+^. Ca^2+^ acts as activator, while Cu^2+^as inhibitor. **(B)** K_m_ and V_max_ values calculated based on the electric current traces, confirm the direct observations of the metal ion influence on proteolytic digestion. **(C)** Digestion of Tat in the presence of Ca^2+^ and Cu^2+^ in the function of time. **(D)** Digestion of Tat in the presence of Ca^2+^ and Cu^2+^ as a function of ionic strength. Reprinted with permission from Wang et al. (2021). Enzyme Hinders HIV-1 Tat Viral Transport and Real-Time Measured with Nanopores. *ACS Sens* 6, 3781–3788. Copyright 2021 American Chemical Society.

Single molecule methods can also be used as sensors for metal ions. Wang et al. (2021) showed that Ca^2+^ and Cu^2+^ ions could be detected by observing how the first enhances and the second inhibits the enzymatic hydrolysis of Tat peptide by trypsin. The passage of the peptide through alpha hemolysin pore is easily detected, whereas hydrolyzed fragments were not registering. This approach also allowed to establish the enzymes parameters—*K*_m_ and *V*_max_ and their changes in presence of one of the metal ions (Wang et al., 2021). To understand the experimental setup and inhibitor role of Cu^2+^, refer to Figure 5.

Similarly, alpha hemolysin nanopore was also utilized for detection of Cu^2+^ ions (Wei et al., 2018). Here passing of the planar tetrapyrrolic macrocycle (TPPS) through the channel resulted only in short lived and low amplitude events. In contrast when Cu^2+^ was added the resulting complex could be detected by observing long lived, high amplitude events. This was caused by Cu^2+^ inducing a distortion of TPPS that results in a molecule with larger space. By careful selection of the chelator molecule the interference from the sample with other metal ions mixed in was not detected. This method can also be used to study interactions between biomolecules and metal ions, as substances forming weak complexes will produce far less meaningful signals than strong complexes.

## 7 Summary

A vast array of biomacromolecules are known for having the capacity to bind metal ion, and their individual study has very important implications in, for example, the realm of health. Previously, this topic has been studied through the usage of bulk methods, nevertheless, it has been shown that they pose some limitations which are surpassed by single molecule characterization, like smFRET, nanopores, magnetic tweezers and optical tweezers. While each individual single-molecule method has limitations of their own, they prove to be extremely useful and applicable methods in characterizing metal ion binding biomacromolecules. Therefore, there is still much to learn about metal ion-binding biomacromolecules, and the single-molecule approaches are valuable methods of study. Nevertheless the application of them seems to be limited as only a several publications appear on the subject each year. This could be attributed to the specific measurement conditions described above. That being said it is our opinion that the possible gain of knowledge on metal ion binding biomolecules combined with constant single molecule method development will popularize their usage in this field in future.
